# Overexpression of *Plasmodium berghei* ATG8 by Liver Forms Leads to Cumulative Defects in Organelle Dynamics and to Generation of Noninfectious Merozoites

**DOI:** 10.1128/mBio.00682-16

**Published:** 2016-06-28

**Authors:** Christiane Voss, Karen Ehrenman, Godfree Mlambo, Satish Mishra, Kota Arun Kumar, John B. Sacci, Photini Sinnis, Isabelle Coppens

**Affiliations:** a Department of Molecular Microbiology and Immunology, Malaria Research Institute, Johns Hopkins University Bloomberg School of Public Health, Baltimore, Maryland, USA; b Division of Parasitology, CSIR-Central Drug Research Institute, Lucknow, UP, India; c Department of Animal Biology, School of Life Sciences, University of Hyderabad, Hyderabad, India; d Department of Microbiology and Immunology, University of Maryland School of Medicine, Baltimore, Maryland, USA

## Abstract

*Plasmodium* parasites undergo continuous cellular renovation to adapt to various environments in the vertebrate host and insect vector. In hepatocytes, *Plasmodium berghei* discards unneeded organelles for replication, such as micronemes involved in invasion. Concomitantly, intrahepatic parasites expand organelles such as the apicoplast that produce essential metabolites. We previously showed that the ATG8 conjugation system is upregulated in *P. berghei* liver forms and that *P. berghei* ATG8 (PbATG8) localizes to the membranes of the apicoplast and cytoplasmic vesicles. Here, we focus on the contribution of PbATG8 to the organellar changes that occur in intrahepatic parasites. We illustrated that micronemes colocalize with PbATG8-containing structures before expulsion from the parasite. Interference with PbATG8 function by overexpression results in poor development into late liver stages and production of small merosomes that contain immature merozoites unable to initiate a blood infection. At the cellular level, PbATG8-overexpressing *P. berghei* exhibits a delay in microneme compartmentalization into PbATG8-containing autophagosomes and elimination compared to parasites from the parental strain. The apicoplast, identifiable by immunostaining of the acyl carrier protein (ACP), undergoes an abnormally fast proliferation in mutant parasites. Over time, the ACP staining becomes diffuse in merosomes, indicating a collapse of the apicoplast. PbATG8 is not incorporated into the progeny of mutant parasites, in contrast to parental merozoites in which PbATG8 and ACP localize to the apicoplast. These observations reveal that *Plasmodium* ATG8 is a key effector in the development of merozoites by controlling microneme clearance and apicoplast proliferation and that dysregulation in ATG8 levels is detrimental for malaria infectivity.

## IMPORTANCE

Malaria is responsible for more mortality than any other parasitic disease. Resistance to antimalarial medicines is a recurring problem; new drugs are urgently needed. A key to the parasite’s successful intracellular development in the liver is the metabolic changes necessary to convert the parasite from a sporozoite to a replication-competent, metabolically active trophozoite form. Our study reinforces the burgeoning concept that organellar changes during parasite differentiation are mediated by an autophagy-like process. We have identified ATG8 in *Plasmodium* liver forms as an important effector that controls the development and fate of organelles, e.g., the clearance of micronemes that are required for hepatocyte invasion and the expansion of the apicoplast that produces many metabolites indispensable for parasite replication. Given the unconventional properties and the importance of ATG8 for parasite development in hepatocytes, targeting the parasite’s autophagic pathway may represent a novel approach to control malarial infections.

## INTRODUCTION

Malaria parasite species that infect humans must first take up residence in hepatocytes before invading red blood cells, which initiates the pathology associated with malaria. *Plasmodium* sporozoites are deposited in the host skin by infected *Anopheles* mosquitoes. Early events in the biology of sporozoite infection have been well investigated, including the transmigration through tissues and invasion of hepatocytes (1, 2). However, much less is known about the events that occur after hepatocyte invasion, beginning with the phenotypic transformation of sporozoites into trophozoites. A key to the parasite’s successful intracellular development in the liver is the morphological and metabolic changes required for the sporozoite-to-trophozoite conversion. We previously showed that concomitantly with their change in shape from elongated sporozoite to round trophozoite, converting parasites expel their micronemes, organelles needed for motility or invasion and thus useless for parasite replication (3). Autophagy is the archetypal disposal pathway for keeping the cell interior clean of disused and superfluous organelles (4). We illustrated that parasites sequester micronemes into double-membrane vesicles resembling autophagosomes, suggesting that an autophagy-like process is activated during parasite conversion in the liver.

In contrast to mammalian cells and yeast that contain ~40 autophagy-related genes (ATG), the malaria parasite contains ~15 orthologs of ATG identified by *in silico* comparative studies: those whose products are required for vesicle expansion and completion are present, while genes involved in induction of autophagy and cargo packaging are mostly absent (5–9). All the components of the ubiquitin-like ATG8 system, which are involved in autophagosome formation, are expressed by *Plasmodium*. We previously cloned and characterized the single *Plasmodium berghei* orthologs of ATG8, ATG3, and ATG7 (10). Interestingly, coexpression of PbATG8, PbATG3, and PbATG7 is upregulated during early sporozoite differentiation in liver cells. In liver forms of *P. berghei*, PbATG8 localizes to a network of vesicles and tubules that align along the apicoplast, a relict plastid organelle, as well as to numerous vesicles in the cytoplasm (10, 11). PbATG8 is preferentially distributed on the outermost membranes of the apicoplast (10), which are enriched in phosphatidylinositol 3-phosphate (PI3P) (12), a phospholipid that marks autophagic structures. In mammalian cells, PI3P acts as a membrane-bound localized lipid signal that controls membrane dynamics by recruiting cytosolic effectors that mediate membrane deformation, expansion, and vesicle transport (13). In many systems, various organelles can supply the nascent autophagosomes with lipids, depending on physiological and pathological conditions (14–16). Based on our observations, we hypothesize that *Plasmodium* liver forms may exploit the ATG8 conjugation pathway to mediate the elimination of unwanted organelles such as micronemes by autophagy and, that the apicoplast may be the lipid provider to generate autophagosomes sequestering micronemes. Otherwise, the single ATG8 in *Plasmodium* may perform several functions within the parasite. The mammalian LC3 homologs γ-GABARAP and GATE-16 participate in vesicular transport from the endoplasmic reticulum (ER) to the Golgi apparatus and within the Golgi apparatus (17). PbATG8-labeled vesicles distributed throughout the cytoplasm suggest that PbATG8 also plays such a role in facilitating vesicular transport of lipids to the apicoplast, contributing to the shape change and size expansion of this organelle that occur during the intrahepatic development of *Plasmodium*. Interestingly, ATG8 expressed in the related apicomplexan parasite *Toxoplasma gondii* is also localized to the apicoplast, and it seems to be involved in the maintenance of this organelle and proper segregation between daughter cells during replication (18).

The aim of this study was to provide additional insights into the deconstruction of the sporozoite invasion apparatus (micronemes) in *P. berghei* liver forms and to explore the potential contribution of PbATG8 toward the selective clearance of micronemes. We show that micronemes are sequestered into PbATG8-positive structures that localize with the apicoplast. A mutant parasite line overexpressing ATG8 during liver infection exhibits growth defects and is unable to finalize merozoite formation. At the cellular level, we illustrate that the compartmentalization of micronemes in the cytoplasm of PbATG8-overexpressing parasites is delayed. Concomitantly, the apicoplast undergoes an abnormally fast expansion and fails to be incorporated into the cell of nascent hepatic merozoites. This suggests a dual role for PbATG8 in the coordination of organelle homeostasis, e.g., microneme clearance and apicoplast expansion in liver forms.

## RESULTS

### *Plasmodium falciparum* infecting human hepatocytes in a chimeric mouse expresses ATG8 until the merozoite stage.

**FIG 1:**
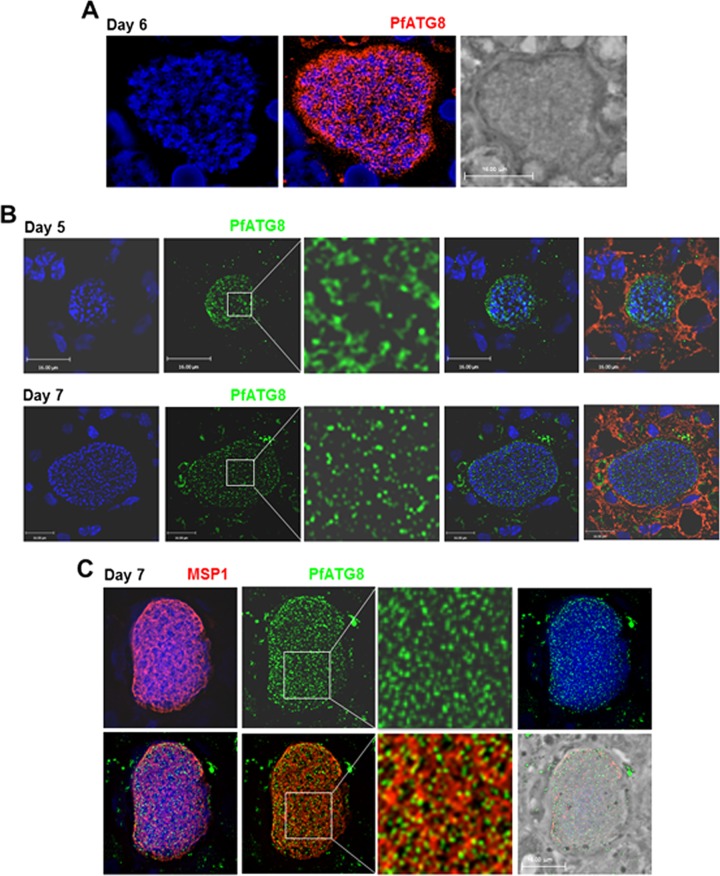
Expression of ATG8 by liver forms of *Plasmodium falciparum* in a humanized mouse. Immunofluorescence assays on mouse liver sections containing *P. falciparum*-infected human hepatocytes using anti-PfATG8 antibodies (red [A] or green [B]) at the indicated days p.i. In panel B, cells were counterstained with Evans blue. In panel C, the parasites were immunostained with antibodies against PfATG8 (green) and MSP1 for the plasma membrane of hepatic merozoites (red). DAPI (blue) was used for staining nuclei in all panels. The vast majority of the PfATG8 signal was associated with the PV. Some surrounding liver cells were also reacting with the anti-PfATG8 antibody: nonspecific background staining is inherent in liver tissue due to cell necrosis and high intracellular protein content. Bars, 16 μm.

Our previous immunofluorescence assays (IFA) on *Plasmodium berghei* in hepatoma cells labeled with anti-PbATG8 antibodies illustrated that the parasite expressed PbATG8 on tubulo-vesicular structures (10). Double immunofluorescence staining of *P. berghei* liver forms for PbATG8 and acyl carrier protein (ACP), a resident protein of the apicoplast, revealed that several PbATG8-containing structures were aligned with the apicoplast, and immunoelectron microscopy (immuno-EM) gold staining showed the association of PbATG8 with the two outermost membranes of the apicoplast. The localization of PbATG8 to the apicoplast was partial (but not exclusive) because PbATG8-associated gold particles were also detected on unidentified tubules and vesicles in the cytoplasm. To assess the physiological relevance of our studies for human malaria, we have examined the expression and localization of ATG8 in *Plasmodium falciparum* liver forms (10). In cultured human hepatocytes, a transgenic strain of *P. falciparum* engineered for ectopic expression of mCherry-PfATG8 (*P. falciparum* ATG8) expressed the chimeric protein on tubular structures, in a pattern similar to that observed for PbATG8 in *P. berghei* (10). To examine whether ATG8 could also be expressed by *P. falciparum* in the human liver, we exploited a humanized chimeric mouse containing functional human hepatocytes as an alternative for modeling the *in vivo* interaction of *P. falciparum* parasites and human hepatocytes (19, 20). Human liver-chimeric mice were infected with *P. falciparum* sporozoites and sacrificed 5, 6, or 7 days postinfection (p.i.) to monitor PfATG8 expression by IFA during a liver infection (Fig. 1). Microscopic observations of the parasitophorous vacuole (PV) at day 6 p.i. illustrate a strong signal for PfATG8 within the parasite, evidencing the endogenous expression of the protein by *P. falciparum* in vivo (Fig. 1A). The pattern of PfATG8-containing structures underwent morphological changes: for example, the PV at day 5 p.i. showed a tubular staining while at day 7 the signal became more punctate (Fig. 1B). The shape transformation of PfATG8-containing structures was reminiscent of the dynamic changes of the apicoplast: during parasite replication, the apicoplast exists as a highly dynamic tubular network that undergoes constriction and fission by the end of schizogony to ensure the proper distribution of this organelle into progeny (21). Costaining of PV sections with antibodies against PfATG8 and MSP1, a protein that marks the plasma membrane of hepatic merozoites, identified PfATG8 puncta within the membranous staining of MSP1. This reveals the association of PfATG8 with intracellular structures, and based on the fluorescence pattern, those could correspond to profiles of the apicoplast as observed for PbATG8 (Fig. 1C).

### Micronemes codistribute with PbATG8-containing structures in converting *P. berghei* to liver forms.

**FIG 2:**
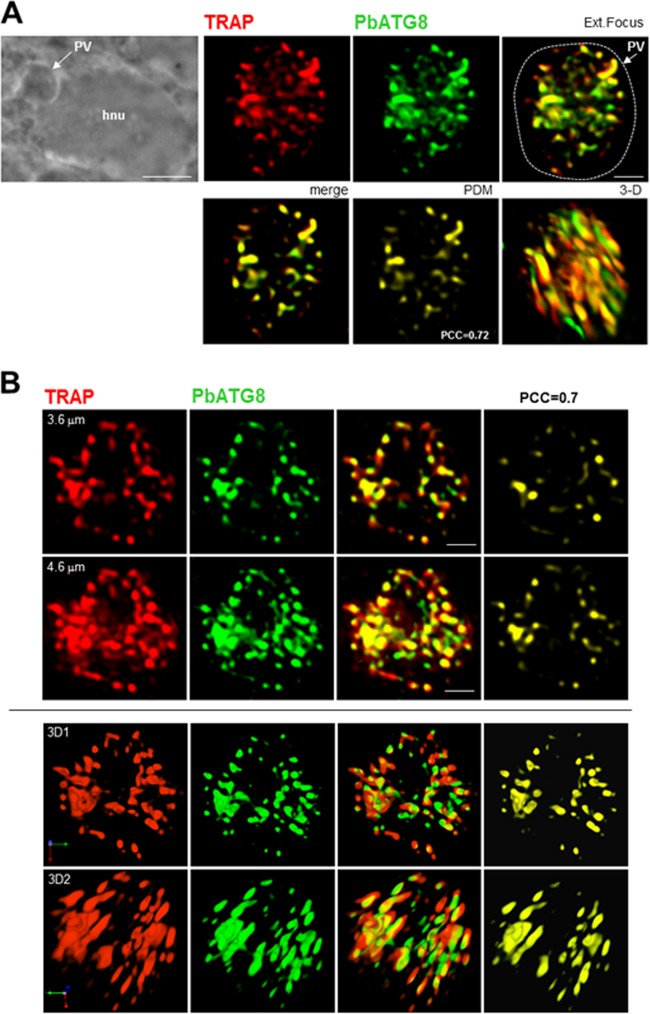
Distribution of micronemes relative to PbATG8-containing structures in *P. berghei*-infected Hepa 1-6 cells. Double IFA on PV using antibodies against PbATG8 (green) and TRAP (red) to label micronemes 17 h p.i. (A) Shown are the phase image at low magnification showing the PV in a Hepa 1-6 cell (hnu, host nucleus), the extended-focus image (a merged image displaying the brightest point of all z-slices in a volume, thus providing maximum depth of field for the regions of the image), the merge image plus the positive product of the differences from the mean (PDM), and a 3-D rotated view of a representative PV. (B) Two optical z-slices of a PV and the 3-D reconstructions of the z-stacks of the same PV are shown. PCCs were calculated from 3 independent parasite preparations. Bars, 1 µm (except for the phase image in panel A, for which the bar is 7 µm).

In converting *P. berghei* in hepatic cells, micronemes are compartmentalized within membrane-bound structures from 10 h p.i., as assessed by IFA and immuno-EM using antibodies against TRAP, a micronemal protein expressed by sporozoites (3). By 20 h p.i., TRAP-labeled structures accumulated at the parasite periphery, while at 36 h p.i., they were detected in the PV lumen, suggesting exocytosis of micronemes. We showed that components of the ATG8 conjugation system in *P. berghei*, PbATG3, PbATG7, and PbATG8, were upregulated at the transcriptional level upon liver infection, suggestive of activation of autophagy in liver forms (10). To examine whether converting parasites discard micronemes using their autophagic machinery, we performed double staining for TRAP and PbATG8 in parasites during liver infection. A significant overlap between TRAP- and PbATG8-labeled structures was observed in the parasite 17 h p.i. (Fig. 2A and B). The level of colocalization between the two fluorescent proteins determined by calculating the product of the difference from the mean (PDM) indicated a mean Pearson coefficient *r* of 0.68 ± 0.15 (Pearson correlation coefficient [PCC] of 0.72 in PV shown in Fig. 2A). Three-dimensional (3-D) reconstructions of optical z-stack sections of the PV shown in Fig. 2A illustrate the remarkable intricacies of PbATG8-labeled structures with clustered micronemes (Fig. 2B). This suggests the involvement of ATG8 in mediating microneme clearance.

### *P. berghei* liver forms generate amphisomes containing PbATG8, PbGRASP, and PbVSP4.

**FIG 3:**
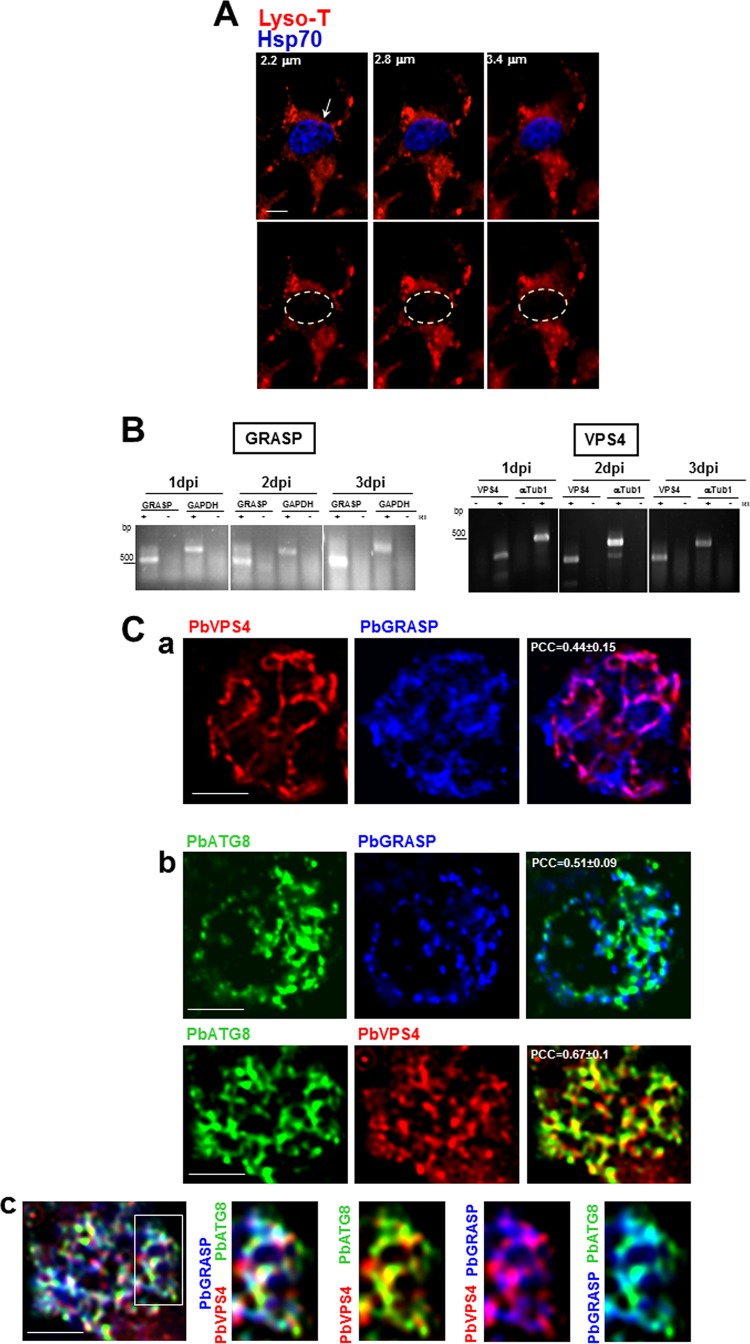
Coassociation of PbATG8 with GRASP- and VPS4-positive structures. (A) Fluorescence assays on *P. berghei*-infected cells stained with LysoTracker (red) 40 h p.i. Parasites were identifiable by immunolabeling for Hsp70 (blue). Three optical z-slices of a PV are shown. Bar, 5 µm. (B) Transcriptional profiles of *GRASP* and *VPS4* in *P. berghei* liver stages. Expression in liver-stage parasites at 1, 2, and 3 days p.i. was assayed by RT-PCR. To verify the absence of genomic DNA contamination, RT-PCRs were set up in duplicate with (+) and without (−) reverse transcriptase (RT). GAPDH or α-tubulin (αTub1) was used as an internal control. PCCs were calculated from 3 independent assays. (C) Double (a and b) or triple (c) IFA on *P. berghei*-infected cells 30 h p.i. using antibodies against PbATG8, PbGRASP, and PbVPS4. PCCs were calculated from 3 independent parasite preparations (*n* = 17 to 26 PVs). Bars, 10 µm.

In eukaryotic cells, autophagosomes can fuse either with lysosomes for intracellular degradation, i.e., autophagy *sensu stricto* (22–25), or with endosomal multivesicular bodies (MVB) to form amphisomes that subsequently fuse with the plasma membrane for release of their content extracellularly, i.e., secretory autophagy or exophagy (26–32). So far, no evidence exists for the presence of lysosomal or degradative compartments in *Plasmodium* liver forms. Upon incubation of *P. berghei*-infected hepatoma cells with LysoTracker, no fluorescent signal was detectable in the parasite, in contrast to the bright fluorescent structures visible in the host cell (Fig. 3A). Thus, in the absence of intracellular acidic lysosome-like organelles, a process of degradative autophagy in intrahepatic *Plasmodium* seems unlikely. Besides, the codistribution of micronemes with PbATG8-positive structures followed by the discharge of micronemes into the PV favors the existence of a process of secretory autophagy operational in the parasite to dispose of micronemes.

In many organisms, secretory autophagy usually arises from cell rearrangements during differentiation (33). This form of autophagy requires the activity of the peripheral Golgi reassembly and stacking protein (GRASP) (34), which binds to autophagosomes prior to fusion with MVB to form amphisomes (33, 35, 36). The genome of *Plasmodium* parasites contains one GRASP homolog, and its predicted sequence harbors the conserved motifs for transoligomerization and membrane anchoring, found in GRASP orthologs (34). *Plasmodium* GRASP has been characterized in the intraerythrocytic forms of *P. falciparum*, and the protein localizes to dispersed vesicles in the parasite cytoplasm (37). The biogenesis of MVB and the sequestration of proteins destined for degradation, recycling, or release in their intraluminal vesicles are driven by the highly conserved ATPase vacuolar protein sorting 4 (VPS4) (38). We previously characterized the VPS4 homolog in *P. falciparum* that mediates MVB formation in blood forms (39). So far, no studies have been undertaken on the presence and morphology of the Golgi apparatus and MVB in *Plasmodium* liver stages. Reverse transcriptase PCR (RT-PCR) showed the transcription of the GRASP and VPS4 genes in *P. berghei* from day 1 to day 3 post-liver infection (Fig. 3B). Moreover, IFA revealed that PbGRASP and PbVPS4 were expressed in *P. berghei* liver forms as assessed 30 h p.i. Parasites exhibited a positive signal on well-defined structures containing PbGRASP and PbVPS4, with partial overlap between these proteins, consistently observed between PV and statistically significant (Fig. 3C, panel a). To examine whether the autophagic pathway may intersect with PbGRASP- or PbVPS4-containing vesicles, we performed IFA for PbATG8 and with either PbGRASP or PbVPS4. Microscopic observations revealed that PbATG8 was partially associated with PbGRASP- and PbVPS4-containing vesicles (Fig. 3C, panel b). We then examined whether parasites can form amphisomal structures containing PbGRASP, PbVPS4, and PbATG8. The triple IFA showed the presence of PbVPS4- and PbGRASP-positive autophagic compartments (Fig. 3C, panel c). These observations point to a potential cross talk between the autophagy machinery and endocytic-exocytic organelles, which may function in organelle disposal by liver forms.

### Generation of a *P. berghei* line with a modified ATG8 3′ untranslated region (UTR) resulting in PbATG8 overexpression.

We hypothesized that PbATG8 plays a role in microneme elimination by exophagy in intrahepatic *Plasmodium*. To evaluate the involvement of *Plasmodium* ATG8 in this process, we attempted to generate an ATG8-knockout mutant strain of *P. berghei*. However, three independent attempts yielded no parasite positive for homologous recombination at the 3′ crossover site, as analyzed by nested PCR, which suggests that PbATG8 is likely essential for the parasite’s survival during blood-stage development (data not shown). We then generated a stage-conditional mutant of *ATG8* using the FLP/FLP recombination target (FRT) site-specific recombination system in yeast (40), adapted for *P. berghei* (41, 42). This method involves the insertion of FRT sites on either side of a genetic element, targeting this region for excision upon expression of the flippase recombinase enzyme (FLP). Following replacement of the endogenous copy of *ATG8* with flippase recognition site (FRT)-flanked *ATG8* introduced into the UIS4/FLP(−) clone of *P. berghei*, expression of the FLP occurs under the control of the UIS4 promoter, which is specific for the salivary gland sporozoite stage. This allows normal expression of PbATG8 in blood stages but FLP/FRT-mediated excision that results in silencing PbATG8 function when sporozoites reach the salivary glands.

**FIG 4:**
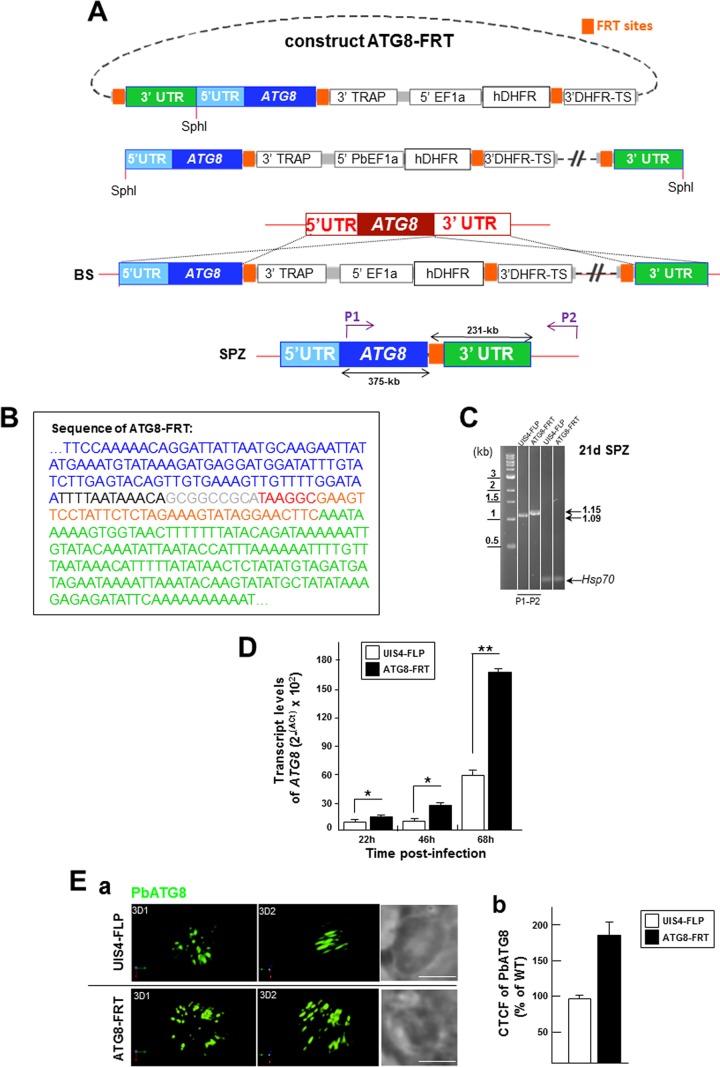
Generation of a parasite strain expressing ATG8 with altered 3′ UTR. (A) Schematic representation of the construct ATG8-FRT. (B and C) The plasmid pUC18-p3′TRAP-hDHFR containing two FRT sites was used as the template for insertion of *ATG8* and the UTRs before linearization with SphI and transfection into a UIS4-FLP parasite strain to generate a parasite strain expressing PbATG8 with a modified 3′ region, which was characterized by the presence of extra nucleotides inserted before the 3′ UTR of *ATG8* as shown in panel B (blue, end of the ATG8 gene; black, 12 bp of 3′ UTR of TRAP; gray, 9 bp of NotI site; red, 6 bp from p3′TRAP-hDHFR flirted; orange, 34 bp from the FRT site; green, beginning of the 3′ UTR of *ATG8*) and confirmed by PCR, as shown in panel C in UIS4-FLP (parental) and ATG8-FRT (3′ mutant) strains. The P1/P2 primers were used to verify the presence of extra nucleotides in the ATG8-FRT strain. The expected sizes of the fragments are shown to the right of the gels. SPZ, sporozoites. (D) ATG8 gene expression measured by qRT-PCR. The α-tubulin 1 gene was used as a reference to normalize the amounts of the ATG8 transcripts. Transcript levels were represented as 2^−ΔΔ*CT*^ × 100 to show levels of transcripts expressed comparatively in the parental and mutant strains. (E) (a) IFA on *P. berghei*-infected cells using anti-PbATG8 antibodies (green) 17 h p.i. Shown are two rotated views of a PV from the parental and mutant strains. Bars, 4 µm. (b) Quantification of PbATG8 fluorescence levels. The corrected total cell fluorescence (CTCF) level was calculated from PVs with wild-type (WT), UIS4-FLP, and ATG8-FRT parasites immunostained for PbATG8 (*n* = 23 to 31 PVs), and data for the two strains are expressed as percentages of wild-type fluorescence levels.

To bypass the lethality problem of blood forms lacking *ATG8*, we created stage conditional knockout (cKO) sporozoites in which the ATG8 gene is preserved with its expression driven by the TRAP 3′ UTR in blood-stage parasites and the original 3′ UTR of the ATG8 gene is interrupted by the plasmid backbone. Upon excision of the *TRAP* 3′ UTR in mosquito salivary glands, the plasmid backbone separates the ATG8 open reading frame (ORF) from its 3′ UTR and ensures abrogation of its function (41). In our cloning strategy described in the Fig. 4A legend, another FRT site was incorporated between the plasmid backbone and the *ATG8* 3′ UTR. Upon excision, the plasmid backbone was removed, allowing the restoration of the *ATG8* 3′ UTR in the UIS4/FLP(−) clone of *P. berghei* but with an extra 60-bp sequence inserted between the ATG8 ORF and its 3′ UTR, as determined by sequencing (Fig. 4B) and verified by PCR (Fig. 4C).

In *Plasmodium*, the majority of the DNA regulatory elements and translational control elements lie in the 3′ UTR, at 1 to 500 bases away from the stop codon of the gene (43). Previous studies reported that genetic manipulation of 3′ UTR impacts gene expression levels (44, 45). We carried out quantitative RT-PCR to compare the transcriptional levels of *ATG8* in cKO parasites (ATG8-FRT strain) and the parental parasites (UIS4-FLP strain) of *P. berghei* liver forms, relative to α-tubulin transcripts (Fig. 4D). Compared to the parental strain, ATG8-FRT parasites expressed significantly higher levels of ATG8 transcripts, corresponding to 1.5-fold, 2.1-fold, and 2.9-fold increases at 22, 46, and 68 h p.i., respectively. Elevated ATG8 mRNA levels in ATG8-FRT parasites compared to those in control parasites could be due to an increase in message stability caused by the insertion in a “GCGGCCGC” sequence among the 60-bp inserted upstream *ATG8* 3′ UTR. Most RNA regulatory elements carry out their functions by inter- or intramolecular base pairing, and it has been reported previously that an increase in GC content in UTRs, especially in an AT-rich genome, results in RNA with more-stable secondary structures (46), as documented for several Hsp genes in many *Plasmodium* species (47).

To monitor the expression levels of PbATG8 in our strains, we performed IFA with anti-PbATG8 antibodies in parasites grown in hepatoma cells for 17 h. Data revealed more abundant structures labeled for PbATG8 in ATG8-FRT parasites than in UIS4-FLP parasites (Fig. 4E, panel a). We next measured the total cell fluorescence intensity of PbATG8 in these strains compared to wild-type parasites. Quantification of the PbATG8 profiles indicated corrected total cell fluorescence (CTCF) values associated with PbATG8 that were ~2 times higher in mutant parasites than in parasites from the parental strain (Fig. 4E, panel b). These findings demonstrate that the insertion of extra nucleotides into the 3′ UTR of *ATG8* results in the enhancement of ATG8 expression, at the transcriptional and translational levels.

### ATG8-FRT parasites are unable to initiate a blood-stage infection.

First, we wanted to assess whether overexpression of PbATG8 could impact parasite infectivity and/or pathogenicity. Five thousand ATG8-FRT or UIS4-FLP sporozoites were injected intravenously into mice, which were tested for blood-stage infection by blood smears daily (Table 1). All mice infected with UIS4-FLP sporozoites became patent and displayed detectable blood-stage infection by day 7 (*P* > 0.01). In contrast, none of the animals infected with the mutant population of parasites were patent at day 7, and all mice stayed negative for the course of the experiment. Even with an intravenous injection of a high dose of parasites corresponding to 50,000 ATG8-FRT sporozoites, no subsequent blood-stage infection could be observed. This suggests that, upon ATG8 overexpression, the parasites were unable to induce a blood-stage infection.

**TABLE 1:** Infectivity of Swiss Webster mice with sporozoites from UIS4-FLP and ATG8-FRT strains

Parasite strain	No. of injected sporozoites	No. of infected animals^a^	Avg % parasitemia on day^b^:		
			7	8	17
UIS4-FLP	5,000	3	0.89	3.23	Sacrificed on day 9
	50,000	3	2.96	6.06	
ATG8-FRT	5,000	3	0	0	0
	50,000	3	0	0	0

a Number of infected animals indicates number of animals injected with sporozoite suspension.

b Average percentage of parasitemia of the 3 sporozoite-injected mice based on detection of erythrocytic stages upon examination from days 4 to 17 postinfection (Giemsa-stained blood smear).

### ATG8-FRT parasites poorly develop into late liver stages.

**FIG 5:**
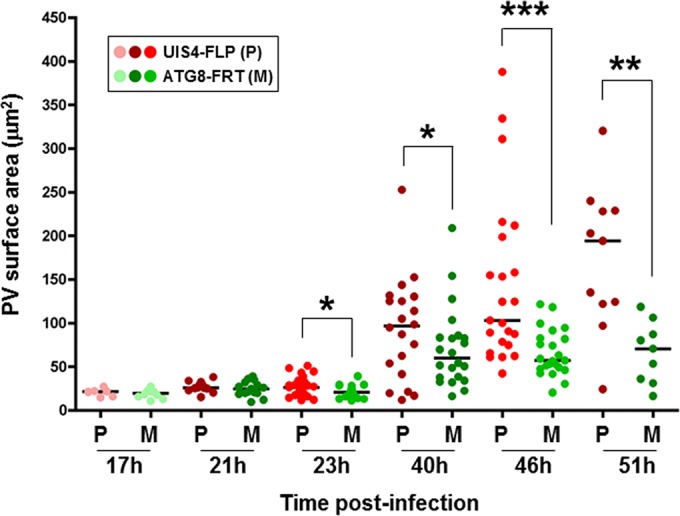
PV size in ATG8-FRT schizonts. Hepa 1-6 cells were infected with parasites from either the ATG8-FRT strain (M, mutant) or the UIS4-FLP strain (P, parental) for the indicated times before fixation. IFA on PV using anti-Hsp70 antibodies was used to identify the PV, and the PV size was measured with Volocity software. Data shown in dot plots are medians from 3 separate biological assays (*n* = 55 to 73 PVs per parasite strain for each time point). *, *P* < 0.0409; **, *P* < 0.0019; ***, *P* < 0.0005 (unpaired *t* test).

Next, we examined whether the failure of mutant salivary gland sporozoites to establish a blood cell infection could be a consequence of impaired development in liver cells. To quantify liver infection, we conducted *in vitro* assays with cultured hepatoma cells infected with ATG8-FRT or UIS4-FLP sporozoites from 17 to 51 h p.i. Parasites were stained with anti-Hsp70 antibodies to score their PV size (Fig. 5). At the onset of infection and until 21 h p.i., the mutant population developed normally, without any significant difference in PV morphology or size from parasites from the parental strain. However, ATG8-FRT parasites significantly suffered a mid-liver-stage developmental defect, more evident from 40 h p.i., while the PV from the parental strain expanded normally in size as the parasites multiplied by schizogony. These data suggest that mutant sporozoites are competent to initiate a liver infection but display a considerable delay in their development and/or maturation in liver cells, which could be held accountable for their ineptness in producing infectious blood forms.

### ATG8-FRT parasites are delayed in the process of microneme elimination.

**FIG 6:**
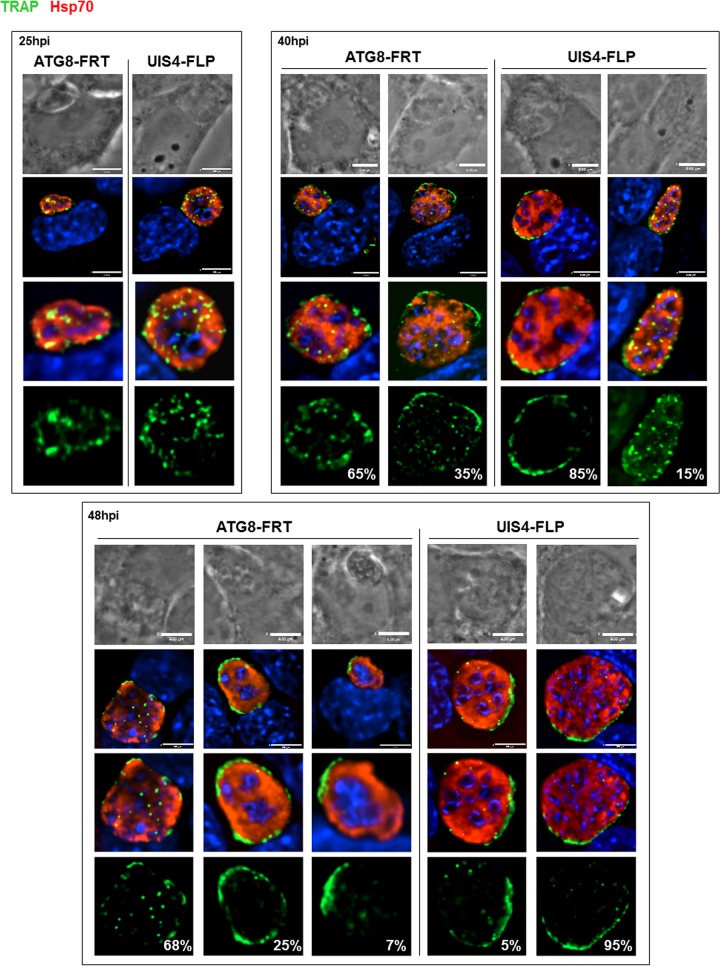
Distribution of micronemes in converting ATG8-FRT parasites. Double IFA of Hepa 1-6 cells infected with either the ATG8-FRT strain or the UIS4-FLP strain for the indicated times using anti-Hsp70 antibodies (red) to identify the PV and anti-TRAP antibodies (green) to monitor the distribution of micronemes during conversion. DAPI (blue) identifies parasite nuclei. The percentage of PVs for a specific TRAP pattern, for either the mutant or the parental strain, at 25, 40, or 48 h p.i. is shown (*n* = 11 to 16 PVs). Bars, 6 µm.

To provide more insights into the significant growth defect observed for ATG8-FRT parasites in liver cells, we scrutinized the organellar content in mutant and parental parasites. We previously showed that micronemes were progressively discarded by the parasite during conversion into liver forms (3). A first examination was made, therefore, to explore the dynamics of micronemes in ATG8-FRT parasites over time by IFA using anti-TRAP antibodies (Fig. 6). UIS4-FLP parasites displayed sequential events comparable to those observed in wild-type parasites (Fig. 2): the clustering of micronemes in the cytoplasm up to 25 h p.i. as illustrated by the presence of very large cytoplasmic TRAP-containing puncta, followed by the expulsion of micronemes into the PV from 40 h p.i. as demonstrated by a circumferential staining of the parasite. Mutant parasites were also able to compartmentalize their micronemes ~25 h p.i. As observed for UIS4-FLP parasites at 25 h p.i., the larger the size of the PV, the greater the TRAP signal that was detected inside the PV. At 40 h p.i., during which the replication of ATG8-FRT parasites starts to slow down, 65% of mutant parasites had retained large intracytoplasmic TRAP-labeled structures, compared to 15% in the parental strain, indicating a delay in microneme evacuation from mutant parasites. Two days p.i., the intracellular TRAP staining was still prominent in 68% of mutant parasites while it looked sparse in all PVs of parental parasites. Despite their aberrant morphology and defects in karyokinesis, mutant parasites that were able to expel micronemes exhibited an extracellular TRAP staining that was either dispersed throughout the vacuolar space (25% of PV) or concentrated at one area of the vacuole (7% of PV).

**FIG 7:**
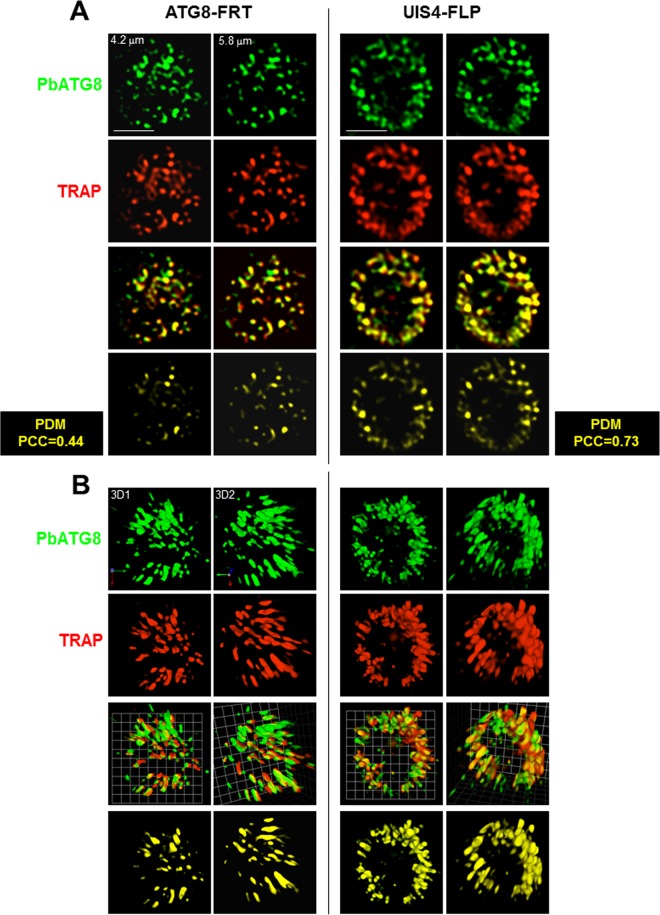
Distribution of micronemes relative to PbATG8-containing structures in ATG8-FRT parasites. (A and B) Double IFA of Hepa 1-6 cells that were infected with either the ATG8-FRT strain or the UIS4-FLP strain for 17 h using antibodies against PbATG8 (green) and TRAP (red). (A) Two optical z-slices of a PV, the extended-focus image, and the image with the positive PDM. (B) Two 3-D rotated views of the PV shown in panel A with the extended-focus image and the image with the positive PDM. Bars, 2 µm.

These observations reveal the poor efficacy of ATG8-FRT parasites for microneme processing and degradation during liver infection. This could be directly due to dysfunctional PbATG8-containing organelles, which are unable to properly sequester micronemes, or indirectly due to the hampered growth of the PV, leading to general weakening of the parasite. As micronemes intersect with PbATG8-containing organelles (Fig. 2), we next wanted to examine whether the delay in microneme clearance could be due to reduced interactions between micronemes and PbATG8-containing organelles. The distribution of TRAP-labeled compartments relative to PbATG8-containing organelles in ATG8-FRT parasites was examined in hepatoma cells infected for 17 h with ATG8-FRT sporozoites (Fig. 7). In UIS4-FLP parasites, a high level of colocalization between the fluorescent signals for TRAP and PbATG8 was observed, with a mean Pearson coefficient *r* of 0.70 ± 0.13 (PCC of 0.73 in PV shown in Fig. 7A), which is close to the values calculated for wild-type parasites (Fig. 2). In comparison, a mean Pearson coefficient *r* of 0.41 ± 0.09 (PCC of 0.44 in PV shown in Fig. 7A) was calculated for the fluorescence signals common to TRAP-labeled compartments and PbATG8-containing organelles in mutant parasites. These differences in overlap between TRAP-labeled compartments and PbATG8-containing organelles in ATG8-FRT versus UIS4-FLP liver forms were more evidently observed on 3-D reconstructions of optical z-stack sections of the PVs shown in Fig. 7A (Fig. 7B). These data illustrating reduced contacts between micronemes and PbATG8 compartments in ATG8-FRT parasites may explain the slower removal of micronemes by mutant parasites.

### The apicoplast expands abnormally fast in ATG8-FRT parasites.

**FIG 8:**
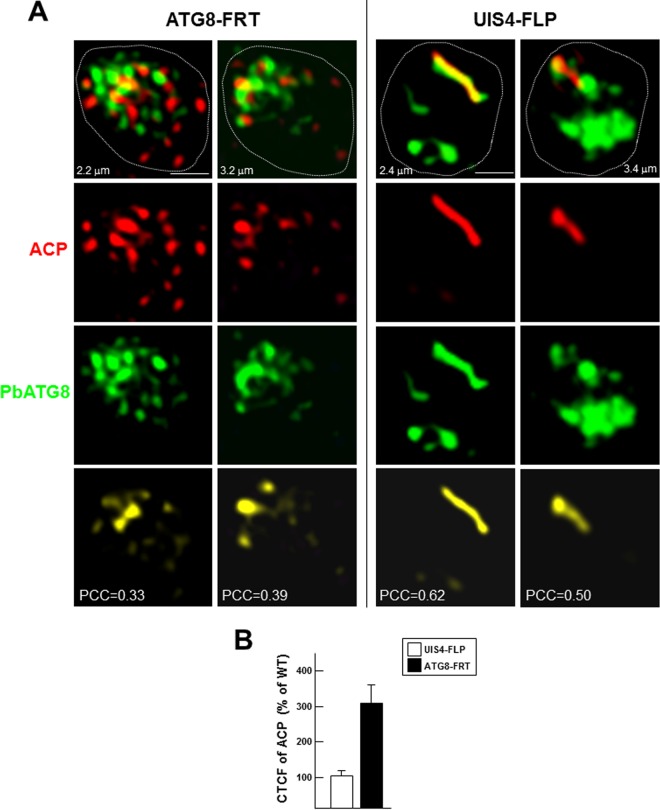
Morphology of the apicoplast in ATG8-FRT trophozoites. Double IFA of Hepa 1-6 cells that were infected with either the ATG8-FRT strain or the UIS4-FLP strain for 9 h using antibodies against ACP (red) for the apicoplast and PbATG8 (green). (A) Two optical z-slices of a PV, the extended-focus image, and the image with the positive PDM. Bars, 1 µm. (B) CTCF levels were quantified on the two strains in reference to wild-type (WT) parasites (*n* = 18 to 25 PVs).

PbATG8 is distributed to the two outermost membranes of the apicoplast in liver forms (10); therefore, its overexpression could result in an altered morphology of the apicoplast, hence affecting proper functions of this organelle. In sporozoite parasites that have just invaded liver cells, the apicoplast starts out as a relatively simple round structure that elongates progressively within the cytoplasm, defining the trophozoite stage. During the schizont stage, the apicoplast branches extensively to ultimately undergo fission such that each daughter merozoite inherits a single small apicoplast (21). To analyze the morphology of the apicoplast and the distribution of PbATG8 in the parasite, hepatoma cells were infected with either ATG8-FRT or UIS4-FLP sporozoites and analyzed by IFA using anti-ACP and anti-PbATG8 antibodies. In early-stage parasites of the parental strain (9 h p.i.), the apicoplast identified by ACP staining had elongated, forming a simple unbranched structure, and PbATG8 was found to be associated with the apicoplast as well as other vesicular structures (Fig. 8A). In sharp contrast, the ACP signal of ATG8-FRT parasites appeared dispersed, suggesting that the apicoplast had remarkably expanded in size by 9 h p.i., forming a large reticulate network. In mutant parasites, PbATG8 was associated with tubulo-vesicular structures, with limited overlap with ACP. Quantification of the ACP signal revealed CTCF values 3 times higher in ATG8-FRT parasites than in control parasites (Fig. 8B).

**FIG 9:**
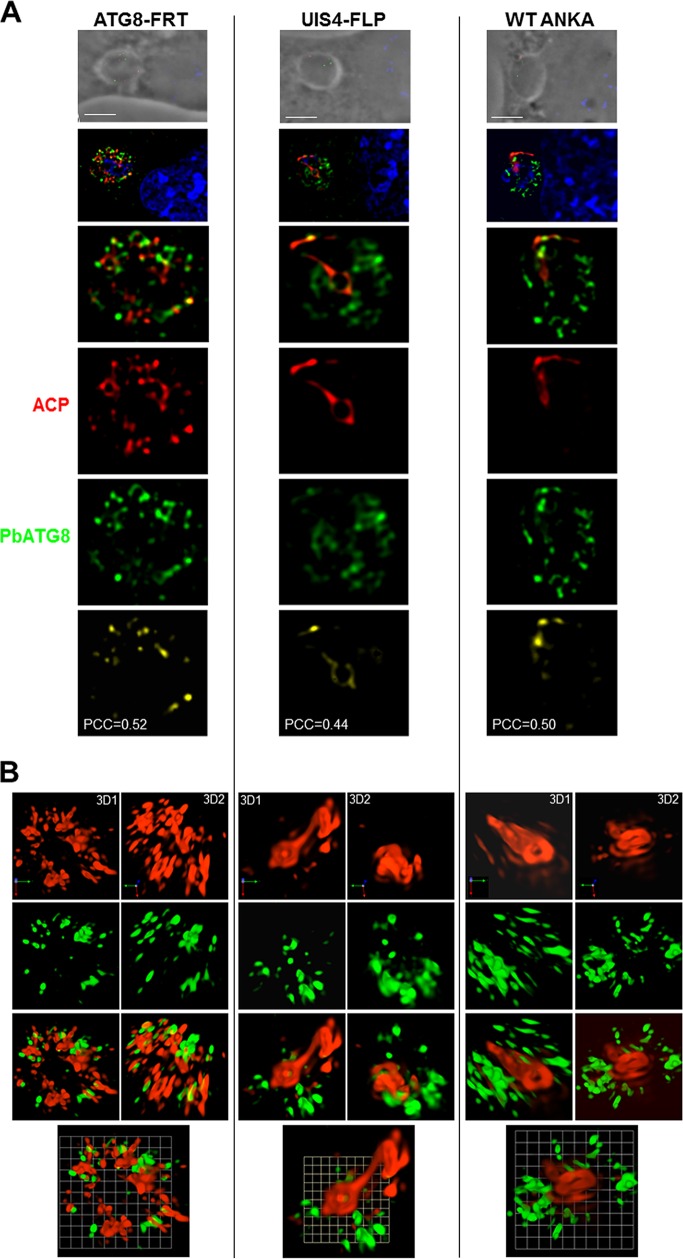
Morphology of the apicoplast in ATG8-FRT young schizonts. (A and B) Double IFA of Hepa 1-6 cells that were infected with wild-type parasites or the ATG8-FRT or UIS4-FLP strain for 17 h using antibodies against ACP (red) and PbATG8 (green). (A) Optical z-slices of a PV at two magnifications, the extended-focus image, the image Bars, 4 microns with the positive PDM, and the phase image. (B) Two 3-D rotated views of the PV shown in panel A with the extended-focus image are represented.

**FIG 10:**
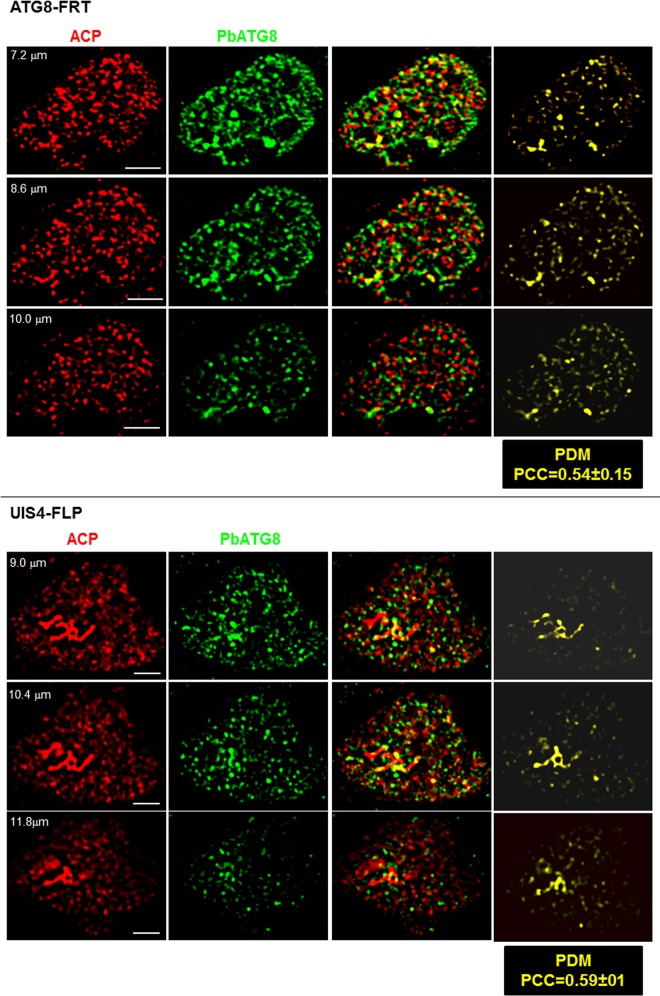
Morphology of the apicoplast in ATG8-FRT late schizonts. Double IFA of Hepa 1-6 cells that were infected with either the ATG8-FRT strain or the UIS4-FLP strain for 40 h using antibodies against ACP (red) and PbATG8 (green). Shown are the three optical z-slices of a PV, the extended-focus image, and the image with the positive PDM. PCCs were calculated from 3 independent parasite preparations. Bars, 4 µm.

At 17 h p.i., in both wild-type and parental parasites, the apicoplast stained for ACP remained a compact structure but started to become branched (Fig. 9A). PbATG8 was associated with the apicoplast, in addition to vesicular structures with various shapes. In contrast, in mutant parasites, the apicoplast formed a large network that aberrantly extended throughout the cytoplasm, with only some spare regions containing PbATG8. These striking morphological differences of ACP-containing structures between control and mutant parasites were more obvious in 3-D reconstructions of optical z-stack sections (Fig. 9B). By 40 h p.i., the apicoplast in early schizonts of parental parasites had transformed into a highly branched, continuous network in control parasites, as seen on serial sections, and PbATG8 was observed abundantly concentrated on intersecting branches (Fig. 10). In mutant parasites, the morphology of the apicoplast was more vesicular than tubular with some distinct spots of fluorescence. Similarly to parental parasites, PbATG8 was predominantly associated with tubular structures stained for ACP, and the mean Pearson coefficients between ACP and PbATG8 were similar in the two strains. Jointly, these observations indicate that, in parasites overexpressing PbATG8, the apicoplast undergoes an unusually fast expansion from the beginning of liver infection, forming in early schizonts a prematurely large network that starts to be dismantled in mature schizonts.

### ATG8-FRT parasites produce small merosomes with poorly differentiated merozoites.

**FIG 11:**
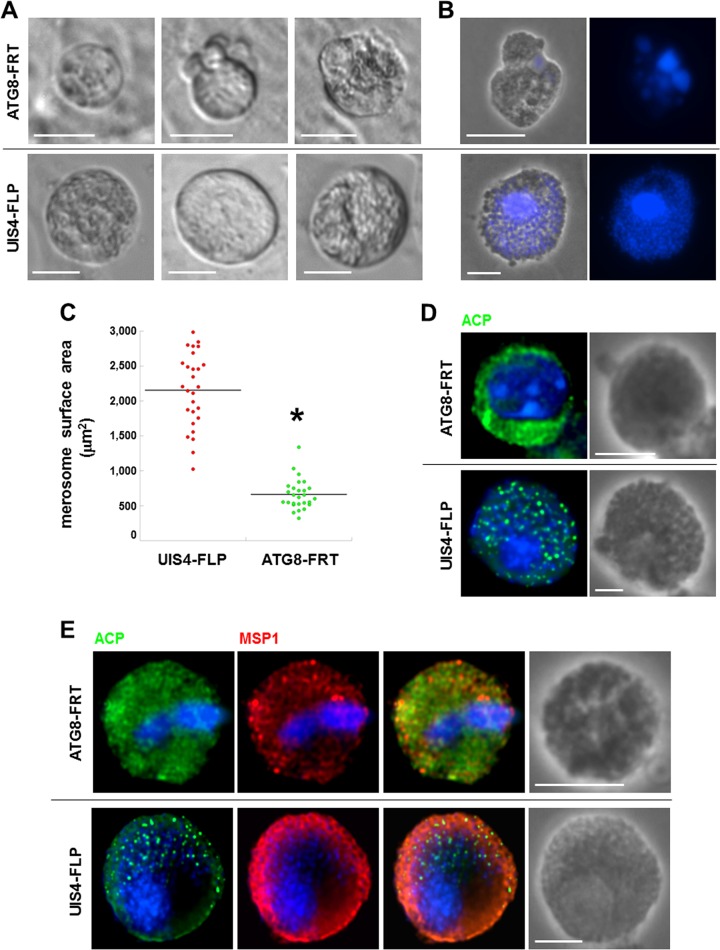
Morphology of ATG8-FRT merosomes. (A) Live microscopy of freshly egressed merosomal structures from Hepa 1-6 cells infected with either the ATG8-FRT strain or the UIS4-FLP strain for 66 to 67 h. Shown are differential inference contrast images of representative merosomal structures from 3 different assays. Bars, 10 µm. (B) DAPI staining of fixed merosomal structures collected 67 h p.i. from Hepa 1-6 cells. Bars, 15 µm. (C) Size distribution of ATG8-FRT and UIS4-FLP merosomes collected in the medium and stained for Hsp70, estimated by using Volocity software. Data shown in dot plots are medians from 3 independent infections (*n* = 12 to 17 merosomes per assay per parasite strain). *, *P* < 0.0005 (unpaired *t* test). (D and E) IFA on ATG8-FRT and UIS4-FLP merosomes immunostained for ACP and MSP1 to visualize parasite cytokinesis 67 h p.i. Bars, 10 µm.

The end of schizogony is characterized by the production of infectious hepatic merozoites enclosed in a membrane derived from the host plasma membrane (48). For a *P. berghei* infection *in vitro*, these parasite-filled structures, so-called merosomes, bud off from host hepatic cells usually from 65 to 72 h p.i., and merosomes, which contain up to hundreds of merozoites, are typically seen moving in the culture medium as buds or free-floating structures. We next assessed the capability of ATG8-FRT parasites to form merosomes containing mature merozoites (Fig. 11). By 67 h p.i., parasites from the parental strain had released spherical merosomes, which were relatively uniform in size with a mean diameter of 18 to 23 µm, as observed for merosomes from wild-type plasmodia (49). Parental merosomes were packed with ~150 merozoites, trackable with 4′,6-diamidino-2-phenylindole (DAPI) staining for individual parasite nuclei (Fig. 11A and B). In contrast to parental parasites, ATG8-FRT parasites could generate ~10-times-fewer merosome-like structures, and they were abnormally small and heterogeneous in shape, and contained poorly discernible individualized merozoites, with nuclear material poorly stainable with DAPI. Quantification of the surface area of the merosomes released by the two parasite strains showed that the merosomes from the parental parasites were up to three times bigger than the ones released by the mutant parasites (Fig. 11C).

**FIG 12:**
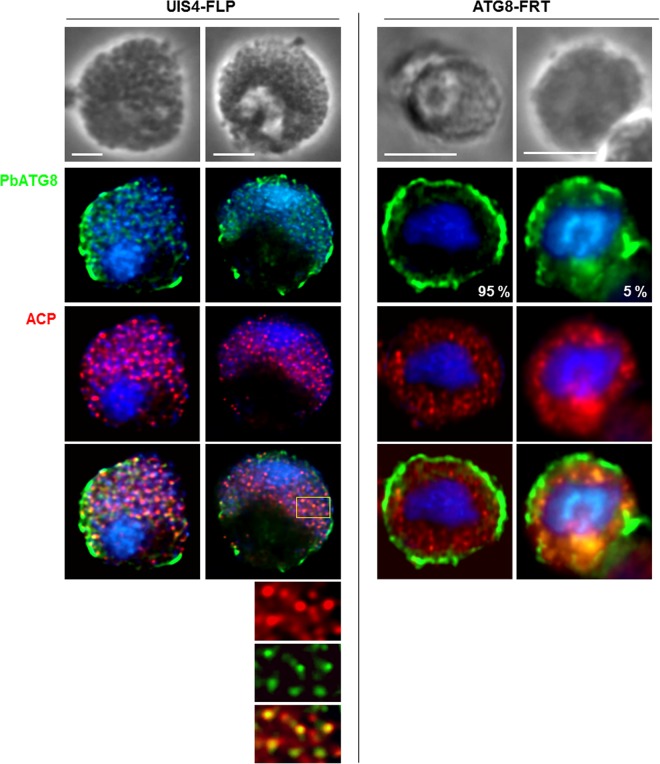
Distribution of PbATG8 in merosomes. Double IFA on ATG8-FRT or UIS4-FLP merosomes collected from Hepa 1-6 cells 67 h p.i. using antibodies against PbATG8 and ACP. The percentage of merosomal structures for a specific PbATG8 pattern in the mutant strain is shown (*n* = 17 PVs). Bars, 10 µm.

We next scrutinized the organellar content and membrane architecture of parasitic forms present in merosomes by IFA. As expected, each hepatic merozoite from the parental strain contained one apicoplast identifiable by a bright fluorescent punctum, labeled for ACP. In contrast, the ACP staining for most of the ATG8-FRT parasites was largely diffuse within the merosomal matrix, suggesting a collapse of the apicoplastic structure (Fig. 11D), or was reduced to a few puncta in less than 5% of merosomes (Fig. 12). ATG8-FRT parasites expressed MSP1; however, the protein was not associated with a distinct membrane demarcating newly formed parasites, as was the case for the parental parasites (Fig. 11E). The data suggest defects in cytokinesis for merozoite generation, as well as improper apicoplast packaging within nascent parasites in the mutant strain.

### ATG8-FRT parasites accumulate PbATG8 at the rim of merosomal structures.

We looked at the localization of PbATG8 in merozoites enclosed in merosomes by IFA. In parental merosomes collected 67 h p.i., the PbATG8 signal was associated with individual apicoplasts stained for ACP (Fig. 12). Interestingly, a PbATG8 staining was also observed at the periphery of the merosomal structures. In contrast, 95% of mutant merosome-like structures accumulated PbATG8 at the rim of merosomal structures. Five percent of mutant merosomal structures exhibited PbATG8 that was, like ACP, diffusely distributed within the merosomal matrix. If PbATG8 is involved in the apicoplast development, defects in apicoplast partitioning in mutant progeny could be due to the disconnection of PbATG8 from this organelle.

### Ultrastructural examinations of the merosomal structures produced by ATG8-FRT parasites reveal abnormal budding of merozoites.

**FIG 13:**
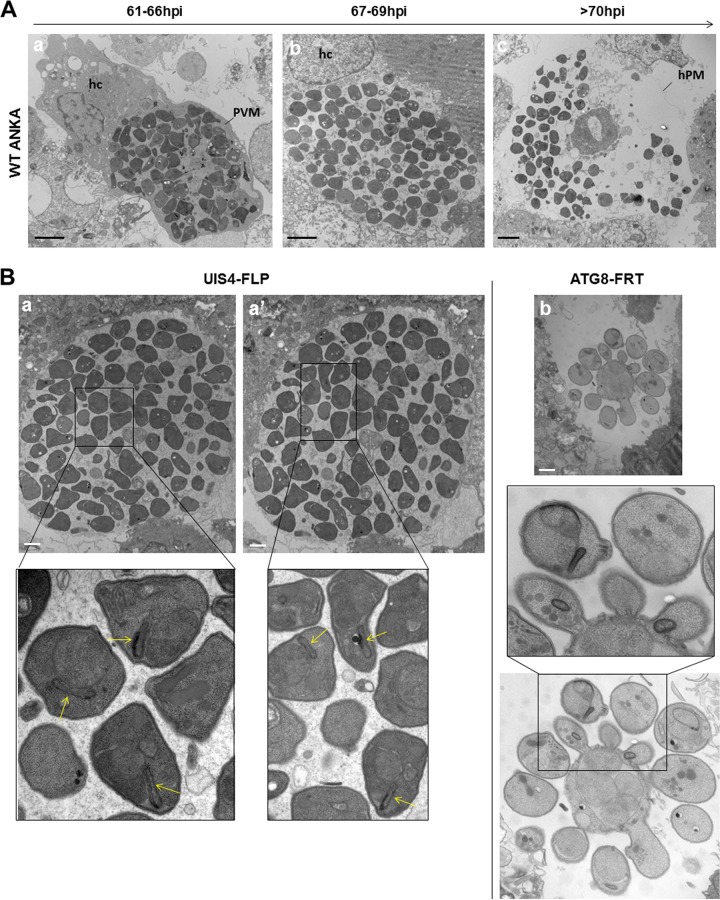
Ultrastructure of ATG8-FRT merosomes. (A) Transmission EM of infected host cell (hc) with wild-type parasites at the indicated times showing normal progression in merozoite formation with parasites surrounded by the PV membrane (PVM) (a) until 66 h that ruptures (b) from 67 h prior to the breakdown of the host plasma membrane (hPM), liberating free merozoites (c) from 70 h p.i. Bars, 5 µm. (B) Transmission EM of infected host cell with either the ATG8-FRT strain or the UIS4-FLP strain 68 h p.i. Panels a and a′ are serial sections of the same merosome from UIS4-FLP parasites showing individual merozoites with apicoplast (arrows in the insets). Panel b illustrates an aberrant merosome-like structure formed by ATG8-FRT parasites, with the image taken at the same magnification as that for UIS4-FLP merosomes shown in panels a and a′. Bars, 2 µm.

The process of hepatic merozoite formation is complex and involves several steps (48). Prior to cytokinesis of *P. berghei* wild type cultivated *in vitro*, parasite nuclei align peripherally along the parasite plasma membrane, which starts to invaginate with a group of nuclei at ~50 h p.i. (cytomere stage I). The parasite membrane continues to invaginate, and ultimately surrounds a single nucleus and a set of organelles necessary for merozoite infectivity (cytomere stage II). Merozoites are fully formed at 61 to 63 h p.i. and remain encased in their PV until 66 h p.i. (merozoite stage III) as seen by EM (Fig. 13A). Shortly after that, the PV membrane ruptures, which liberates parasites into the host cytosol, fenced by the host plasma membrane (merosome stage IV)*.* From 70 h onward, free merozoites that have escaped from merosomes are visible in the culture medium upon loss of host plasma membrane integrity (release stage V).

Our fluorescence microscopic observations show aberrant merosomogony in ATG8-FRT parasites (Fig. 11 and 12). We conducted EM analyses to gain more insights into the defects in merozoite formation and egress from host hepatic cells in the mutant strain. Like wild-type merosomes, those generated by the UIS4-FLP strain at 67 h p.i. were packed with normally constituted merozoites, with less than 2% dead parasites (Fig. 13B, serial sections a and a′). Confirming the ACP labeling in our IFA (Fig. 11 and 12), parental merozoites harbored a typical apicoplast, identifiable by its four membranes. Very few merosomal structures could be collected and processed for EM studies from mutant parasites. Ultrastructural observations on scant mutant merosomal structures 67 h p.i. reveal the presence of parasitic forms budding from a central structure concentrating parasite nuclei, indicative of asynchronized steps forming merozoites (Fig. 13B, panel b). Mutant parasites contained inner membranes and some cytoplasmic organelles, suggesting that they could progress to cytomere stage I, although with considerable delay. In contrast to parental parasites, in which daughter cells budded in a direction perpendicular to the periphery with the conoid facing the parasite plasma membrane, the budding process in mutant parasites was no longer polarized.

### The merosomal structures produced by ATG8-FRT parasites are not infectious.

The buds generated by mutant parasites had overall an abnormal shape and orientation, suggesting that these buds were likely unproductive. To test the capacity of ATG8-FRT parasites to trigger a blood infection, mice were infected with merosomes from mutant and parental strains, and the emergence of blood-stage parasites was monitored daily (Table 2). In three independent experiments, no animals developed a parasitemia after inoculation of 100 ATG8-FRT merosomal structures, compared to mice infected with equal numbers of merosomes produced by UIS4-FLP parasites, which all exhibited a blood infection after 5 days. Even 1,000 ATG8-FRT merosomal structures injected per mouse were unable to establish a parasitic blood infection. Jointly, these findings show that PbATG8 overexpression in *P. berghei* liver forms is detrimental for parasite infectivity in the mammalian host.

**TABLE 2:** Infectivity of Swiss Webster mice with merosomes from UIS4-FLP and ATG8-FRT strains

Parasite strain	No. of injected merosomes	No. of infected animals^a^	Avg % parasitemia on day^b^:		
			5	6	17
UIS4-FLP	100	3	0.38	1.15	Sacrificed on day 7
	1,000	3	2.45	9.13	
ATG8-FRT	100	3	0	0	0
	1,000	3	0	0	0

a Number of infected animals indicates number of animals injected with merosome suspension.

b Detection of erythrocytic stages in the 3 merosome-injected mice upon examination from days 5 to 17 (Giemsa-stained blood smear).

## DISCUSSION

Development and differentiation processes are often accompanied by drastic cellular remodeling that requires organelle degradation and replacement. Autophagy is a fundamental recycling system that produces new building blocks and energy for organelle homeostasis. Like any living organism, *Plasmodium* species undergo continuous cellular renovation that is essential for metabolic adaptations of the parasite to various environments in the vertebrate host and insect vector. In this study, we have investigated the contribution of the single homolog for ATG8 in *Plasmodium berghei*, PbATG8, to organelle turnover and remodeling in liver forms. Transition to a replication-competent metabolically active form in hepatocytes requires for *Plasmodium* both the elimination of superfluous organelles, e.g., micronemes and rhoptries involved in hepatocyte invasion, and the expansion of organelles that provide energy and/or metabolites, such as the endoplasmic reticulum (ER), mitochondrion, and apicoplast (3). Remarkably, micronemes colocalize with PbATG8-containing structures, suggesting the involvement of PbATG8 in microneme processing during differentiation. Of interest, PbATG8 is also detected on amphisomal structures that contain PbGRASP and PbVPS4, and microneme elimination is impaired in *P. berghei* overexpressing PbATG8, which points to a process of secretory autophagy present in liver forms, conceivably to release micronemes. Additionally, PbATG8 localizes in part to the membranes of the apicoplast (10, 11; this study). In *P. berghei* overexpressing PbATG8, this organelle undergoes an abnormally fast expansion, which ultimately leads to a defect in the inheritance of the apicoplast among progeny, which shows a role for PbATG8 in the structural maintenance of the apicoplastic network. Overall, these observations highlight important functions for PbATG8 in controlling the fate and development of organelles, e.g., micronemes and the apicoplast in liver forms.

### Role of PbATG8 in microneme clearance.

Host cell invasion by *Plasmodium* parasites is extremely rapid and relies on a sequence of events that are tightly controlled in time and space. The interaction between the parasite and the host cell is effectuated by micronemal proteins that are released during invasion and that act as ligands for mammalian cell receptors. The selectivity of the host cell to be invaded, e.g., hepatocyte versus erythrocyte, is mediated by specific micronemal proteins that differ from one parasite stage to another: in hepatic and blood merozoites, micronemes contain AMA-1, EBA proteins, ASP, and SUB2, responsible for erythrocyte invasion, while TRAP, SPECT, MAEBL, and PPLP are stored in micronemes of sporozoites and intervene in hepatocyte recognition (50, 51). The degradation of micronemes by the parasite following hepatocyte penetration may then be a strategy to renovate the microneme population and furbish newly formed micronemes with proteins that are required for red blood cell recognition and invasion by liver merozoites.

Intrahepatic *Plasmodium* parasites lack acidic, lysosome-like organelles, which brings into question the capability of the parasite to degrade macromolecules or organelles in intracellular compartments. In the course of their elimination from liver forms, micronemes are clustered and then accumulate near the plasma membrane, and their content (e.g., TRAP) is detected in the PV before disappearing from the parasite. This suggests the expulsion of micronemes from the parasite cell, followed by the extracellular degradation of these organelles. The enzymatic composition of the PV in intrahepatic parasites is not known to assert the degradation of the expelled micronemes in the vacuolar space; however, it is known for blood stages of *P. falciparum* that the PV lumen contains several proteases secreted by the parasite, suggestive of extracellular digestive activities mediated by the parasite (52). The coassociation of PbATG8-containing organelles with clusters of micronemes in the cytoplasm points to a role of *Plasmodium* ATG8 in facilitating microneme clearance from the parasite. The detection of amphisomal structures in liver forms and the upregulation of effectors of the parasite ATG8 conjugation system upon liver infection support the idea of exophagy-mediated activities that are operational during parasite conversion.

Several mammalian cells can undergo dramatic cell renovation during differentiation. A spectacular example is illustrated for nascent erythrocytes in which most of the organellar content is eliminated by autophagy during erythropoiesis (53, 54). At the end of this process, exocytosis of the final bolus of cytoplasmic material involves autophagosomes that combine with endosomes that fuse with the plasma membrane, indicative of secretory autophagic activities in maturing erythrocytes. Mouse genetics provide strong evidence for a central role for autophagy during erythrocyte maturation by showing the persistence of mitochondria in a mouse lacking key autophagy genes, such as *ATG7*, or genes involved in mitophagy, such as *BNIP3L* (55, 56). The ATG4 endopeptidase plays a central role in facilitating autophagosomal maturation, as it primes newly synthesized ATG8 precursor by cleaving at the critical glycine residue, followed by deconjugating (delipidating) lipid-bound ATG8 for recycling (57). Human erythroid cells overexpressing a catalytically inert form of ATG4 paralogs exhibit altered activity of autophagosomes, culminating in the retention of engorged autophagosomes at the reticulocyte stage (54). In these dominant negative ATG4 mutants, ATG8-ATG4 molecules form an abnormally stable interaction, having as consequences a failure in the deconjugation of ATG8 from the outer membrane of the autophagosome, hindrance of ATG8 interactions with other autophagy-related effectors, and/or interference with ATG8-mediated fusion events (58).

Secretory autophagosomes are distinguished from cell-degradative autophagosomes from (i) their cargos, a.o., proinflammatory cytokines (59), acyl coenzyme A (CoA)-binding proteins (ACBP) (60), and α-synuclein (61); (ii) the involvement of amphisomes; and (iii) the participation of GRASP proteins that contribute to the fusion of amphisomes with the plasma membrane (33). In mutants of *Dictyostelium* or yeast lacking GRASP, vesicles containing the acyl-CoA-binding protein Acb1 accumulate near the plasma membrane, underlining the importance of GRASP for Acb1 for the secretory autophagy (27, 28). Not only cytosolic proteins but also nanovesicles, organelles such as autophagosomes and secretory granules, and even pathogens can be released by cells with an engaged autophagic program (29, 62–64). The selective sequestration of micronemes in PbATG8-containing organelles and their release from *Plasmodium* are another example of organelles that undergo secretory autophagy. The reduced interactions between micronemes and PbATG8 compartments with consequences of a longer retention of micronemes in parasites with dysregulated levels of PbATG8 suggest a role for PbATG8 in controlling autophagosome fusion with the plasma membrane. Generation of a parasite line with reduced expression of ATG8 in liver forms will provide additional information on the level of dysregulation in organelle development for liver forms. In mammalian cells, alteration in the expression of ATG4B negatively impacts autophagy activities (65). ATG4B overexpression reduces the amounts of the LC3 lipidated form and affects the formation of LC3-associated autophagosomes, indicating that a disproportionate ratio in autophagy proteins could impair autophagosome maturation. As observed in ATG4B-overexpressing mammalian cells, dysregulated expression of PbATG8 in *Plasmodium* may also induce autophagic activity defects due to abnormal PbATG8 interactions with partners, rendering PbATG8-containing autophagosomes dysfunctional. Hybrid autophagosome/endosome structures containing PbATG8, PbGRASP, and PbVPS4 are formed by intrahepatic parasites. *Plasmodium* GRASP and VPS4 may be involved in the coordination of the exophagic events of micronemes. Generating conditional gene knockout parasites for GRASP and VPS4 would be essential to assessing the physiological relevance of these proteins for liver-form maturation into merozoites.

A precedent example linking autophagy and differentiation in protozoa has been reported for *Trypanosoma brucei*. Like *Plasmodium* parasites, trypanosomes shuttle between an insect vector and a mammalian host, and adaptation to these various environments requires changes in organelle population and metabolic pathways. The bloodstream forms of *T. brucei* depend entirely on glucose metabolism through glycolysis, whose enzymes are compartmentalized in glycosomes and in turn have repressed mitochondrial activities (66). In contrast, insect forms, which need to survive in different regions of the tsetse fly’s digestive system wherein the glucose supply is irregular, rely on mitochondrial metabolism for ATP production and have sparse glycosomes (67). It has been recently shown that bloodstream forms overexpressing ATG8 proteins and incubated in a differentiation medium have a delayed transition to insect forms, as illustrated by a longer retention of glycosomes (68). Similarly to *P. berghei* overexpressing PbATG8, excess ATG8 in *T. brucei* causes a dysregulation in the autophagy flux, resulting in impairment in parasite differentiation and functionality.

### Role of PbATG8 in apicoplast maintenance.

Interestingly, PbATG8 may also be involved in the maintenance of the apicoplast shape. This function is *a priori* distinct from autophagy; however, ATG8 can cause the hemifusion of vesicles *in vitro*, and if this property were related to the membrane expansion of the phagophore (8), it would imply a general role for ATG8 proteins in lipid/vesicle supply for increasing the size of any organelle. In *Plasmodium*, vesicles containing apicoplast-targeted proteins bud from the secretory pathway and then fuse with the outermost membrane of the apicoplast (69, 70). PbATG8 is distributed to numerous vesicles in the vicinity of the apicoplast, and it may be involved in the trafficking and fusion of vesicles with the apicoplast, facilitating organelle expansion during schizogony. In favor of this possibility is the accelerated development of the apicoplastic network in *P. berghei* parasites that overexpress PbATG8. Of interest, the expansion of the ER and mitochondria in T lymphocytes is also developmentally regulated by autophagy. Abnormal growth of these organelles has been observed in ATG7- or ATG3-depleted T cells (71). In these mutant T cells, the disproportionate levels of ATG8 versus ATG3 and ATG7 could mimic a situation of ATG8 overexpression for which a common feature would be loss of control of organelle development.

Nevertheless, the altered architecture of the apicoplast in PbATG8-overexpressing parasites has for an outcome the generation of defective parasites unable to establish a blood infection. Defects in apicoplast morphology most likely lead to a global disorganization of this organelle and thus to functional perturbations. The apicoplast has lost its photosynthetic functionality but has retained a number of important functions, including the biosynthesis of fatty acids, heme, and isopentenyl diphosphate (72). It is well known that interference with these biosynthetic pathways is detrimental for the parasite. For instance, fosmidomycin, which affects isoprenoid biosynthesis (73), and antibiotic agents that act as apicoplast translation inhibitors (74) cause a so-called delayed death phenotype (75). Gene deletion in the type II fatty acid biosynthesis (FASII) pathway leads to parasite premature arrest, as evidenced by the absence of signature proteins of mature blood-stage infectious parasites (76–78). Depletion of the *Plasmodium*-specific apicoplast protein (PALM) results in defects in liver merozoite formation *in vitro* and impaired initiation of a blood-stage infection (79). Overall, these examples emphasize the importance of apicoplast functions for the survival of liver-stage parasites, and currently, the apicoplast is one of the leading targets for novel antimalarial drugs. If PbATG8 is central for apicoplast integrity and the structural maintenance of this organelle, dysregulated expression of this protein may globally affect many apicoplastic functions, resulting in weakening of the parasite by insufficient production of essential metabolites.

In PbATG8-overexpressing parasites, the process of cytokinesis for merozoite cellularization is impaired, as well as apicoplast redistribution between nascent liver merozoites. ATG8 in *Plasmodium* species is ~70% identical to ATG8 in *Toxoplasma gondii*. A role of ATG8 in apicoplast inheritance in growing daughter cells has been also illustrated in *T. gondii*. During the division of the apicoplast in *T. gondii*, each end of the elongated apicoplast remains associated with one of the duplicated centrosomes (80). A recent study shows that TgATG8 plays a role in positioning the apicoplast at the centrosomes during division (18). Down-expression of TgATG8 leads to abnormal segregation of the apicoplast into the progeny, due to a loss of physical interactions of the organelle with the centrosomes.

### Involvement of the apicoplast in autophagosome biogenesis?

The connection between the overproliferation of the apicoplast and defects in microneme expulsion in *Plasmodium* overexpressing PbATG8 suggests that the apicoplast may be the putative source of autophagosomal membranes. This assumption is further reinforced by the association of PbATG8 on the apicoplast with the presence of high levels of PI3P on the membranes of this organelle (12), two molecular markers of autophagosomes in mammalian cells. The main pathway for the synthesis of PI3P involves the phosphorylation of PI on the 3′ position of the inositol ring by class III PI3 kinase or Vps34. In mammalian cells, Vps34-containing vesicles can fuse with the ER, enabling the synthesis of PI3P and the local concentration and activation of PI3P effectors in the PIP3 platforms. These effectors (e.g., Alfy, ATG14, and ATG21) are involved in the nucleation of a preautophagosomal structure that expands into a phagophore (81, 82). The *Plasmodium* genome encoded one predicted Vps34-type enzyme containing the characteristic domains of class III PI3P kinases (83). The expression and localization of *Plasmodium* Vps34 in *Plasmodium* liver forms are still unknown. If this enzyme is localized to the apicoplast, it will be interesting to examine whether it can drive autophagy by initiating the formation of preautophagosomal structures budding off from the membrane of the apicoplast.

### *Plasmodium* ATG8 as drug target.

Owing to the cytoprotective role of autophagy, several human pathologies (e.g., neurodegenerative diseases, infections, and cancers) are linked to defective autophagy that could result from either excessive or diminished ATG protein activities (84). In view of these findings, many pharmacological agents include autophagy activators (e.g., rapamycin) or autophagy inhibitors (e.g., chloroquine) to enhance the protective role or inhibit the destructive role of autophagy in different diseases, respectively. Our data show that PbATG8 is a key effector in the development of infectious merozoites. Mild levels of PbATG8 overexpression (1.5- to 2.9-fold) result in severe defects in the completion of the parasite hepatic cycle and complete failure of parasite overexpressers to enter the symptomatic erythrocytic stage. This reveals the high sensitivity of *Plasmodium* ATG8 to genetic manipulations and augurs a promising outcome for chemotherapeutic intervention targeting the autophagic machinery of *Plasmodium*. Chloroquine, the prototype antimalarial drug, affects *Plasmodium* blood forms by blocking the fusion of endosomes carrying host hemoglobin to the digestive vacuole and inhibiting plasmepsin activity by raising the pH in endocytic compartments (85). The molecular link between chloroquine and autophagy has been established in *Plasmodium falciparum* strains that have become resistant to chloroquine. Upon chloroquine exposure, chloroquine-sensitive *P. falciparum* accumulates PfATG8-labeled puncta in the cytoplasm (86), consistent with an upregulation of autophagosome formation or inhibition of autophagosome fusion. Comparison of the genetic profiles of chloroquine-sensitive and chloroquine-resistant parasites identifies mutations in several autophagy genes, including ATG11 and ATG14 (in addition to the chloroquine transporter PfCQT) (86). In support of this observation, chloroquine-resistant parasites show dysregulation in the autophagic response, as they do not form ATG8 puncta upon incubation in chloroquine, which indicates that they have evolved resistance to chloroquine-induced perturbation in autophagy.

*P. falciparum* in human hepatocytes also expressed ATG8, making our observations on *P. berghei* relevant for human malaria. Better understanding of how *Plasmodium* ATG8 and its partners contribute to the infectivity of the malaria parasite is warranted to identify promising therapeutic targets in *P. falciparum*.

## MATERIALS AND METHODS

### Reagents and antibodies.

All chemicals were obtained from Sigma Chemical Co. (St. Louis, MO) unless indicated otherwise. Antibodies used for immunofluorescence assays included rabbit and rat anti-PbACP (1:800 and 1:1,000 dilutions, respectively) from S. Prigge (Johns Hopkins University); rat anti-PbATG8 (1:200 dilution) (10); mouse anti-PfATG8 (1:100 dilution) (87); rabbit anti-GRASP (1:500 dilution) (88); mouse anti-PfVPS4 (1:150 dilution) (37); rabbit anti-*Plasmodium yoelii* MSP1 (anti-PyMSP1) (1:500 dilution) from MR4 (Manassas, VA); rabbit anti-TRAP (1:300 dilution) from U. Frevert (New York University); and mouse anti-PbHsp70 (1:100 dilution) from F. Zavala (Johns Hopkins University). LysoTracker Red DnD-99 and secondary anti-IgG antibodies conjugated to Alexa^488^ or Alexa^594^ (1:2,000 dilution) were obtained from Invitrogen (Carlsbad, CA).

### Mice.

All animal procedures were approved by the Institutional Animal Care and Use Committee of Johns Hopkins University according to National Institutes of Health guidelines for animal housing and care. Six- to 8-week-old female Swiss Webster mice purchased from Taconic Biosciences, Inc. (Albany, NY), were used for *P. berghei* infection.

### Mammalian cell line and parasite strain.

Mouse Hepa 1-6 cells used to cultivate *P. berghei* parasites were obtained from ATCC (ATCC CRL-1830). Cells were grown as monolayers at 37°C in an atmosphere of 5% CO_2_ in alpha minimum essential medium (α-MEM; Gibco), supplemented with 10% fetal bovine serum (FBS), 2 mM l-glutamine, and 50 µg/ml penicillin/streptomycin. All *P. berghei* ANKA lines (wild-type, parental, and mutant strains) were passaged in *Anopheles stephensi* mosquitoes blood-fed on infected mice as described previously (89).

### *Plasmodium* cultivation and isolation.

Sporozoites isolated from disrupted salivary glands 22 days post-infection (p.i.) of *A. stephensi* mosquitoes were used either to infect Hepa 1-6 cells (for *in vitro* assays) or for injection into the tail vein of mice (for virulence assays). To collect parasite blood stages, blood was withdrawn from infected mice by cardiac puncture using syringes coated with a stock solution of 100 U/ml of heparin to prevent clotting. For merosome production, 1 × 10^5^ Hepa 1-6 cells per well in a 24-well plate were seeded 1 day prior to infection with 5,000 sporozoites isolated from mosquitoes 22 days p.i. and cultivated in medium containing 5 µg/ml amphotericin B (Fungizone) for 68 to 72 h. Merosomes budding from cells or floating in the culture supernatant were harvested after gentle swirling of the plate.

### Virulence of *Plasmodium* strains in animals.

To assess the virulence of sporozoites, six Swiss Webster mice were infected via tail vein injection with 5,000 or 50,000 sporozoites (parental or mutant strains) per animal in three independent experiments. For determination of merosome infectivity, six Swiss Webster mice were infected via tail vein injection with 100 or 1,000 merosomes (parental or mutant strains) per animal in three separate assays. Blood-stage parasitemia was monitored daily by Giemsa staining on thin blood smears on glass slides.

### Generation of PbANKA ATG8-3′ UTR mutant.

The conditional knockout targeting vector was constructed using a plasmid vector as the template consisting of two FLP recognition target sequences (*FRT* sites) flanking the 3′ UTR of the *TRAP* gene and the human dihydrofolate reductase (hDHFR) expression cassette (from R. Menard, Pasteur Institute, France). Two PCR fragments were amplified from the PbANKA wild-type genomic DNA. The first fragment was amplified with the primers UTR-Sph1-r and UTR-HindIII-FRT-f and ligated into SphI and HindIII sites. To amplify the second fragment, the primers c-term-Not1-r and c-term-Sph1-f were used and the resulting PCR product was cloned into NotI and SphI. The plasmid was linearized with SphI prior to transfection into the parent parasite line UIS4/FLP deleter clone as described elsewhere (90, 91; S. Mishra, K. A. Kumar, and P. Sinnis, unpublished data). Successful integration in transfected parasites was confirmed by PCR using the primers Ef1alpha-r and PbAtg8-304-f to verify the 5′ end cassette and the primers PbAtg8+750-r and coko-f to verify the 3′ end cassette. The presence of Hsp70 as the control was verified using primers PbHsp70-r and PbHsp70-f. After limiting dilution of drug (pyrimethamine)-resistant parasites, mice were infected to isolate parasite single clones as described previously (90).

### Transcriptional analysis.

**T**o examine GRASP and VPS4 gene expression, RNA was purified from *P. berghei*-infected Hepa 1-6 cells at days 1, 2, and 3 using the RNeasy RNA purification kit (Qiagen, Valencia, CA). One microgram of total RNA was used to generate cDNA as a template for RT-PCR (Invitrogen) using gene-specific primers synthesized by Integrated DNA Technologies (see Table S1 in the supplemental material). Relative GRASP and VPS4 gene expression was compared to the glyceraldehyde-3-phosphate dehydrogenase (GAPDH) and α-tubulin 1 housekeeping genes of *P. berghei*, respectively. As a negative control, a second sample of RT-minus cDNA was also generated by omission of the reverse transcriptase and used for PCR. To quantify ATG8 gene expression over time by reverse transcriptase quantitative PCR (qRT-PCR), total RNA was isolated from liver forms 22, 46, and 68 h p.i. using the RNeasy minikit (Qiagen). Two micrograms of RNA was reverse transcribed using the SuperScript III First-Strand synthesis system (Invitrogen) with reverse oligonucleotides as gene-specific primers. Five hundred nanograms of cDNA was amplified with the primer pairs qRT_8_f and qRT_8_r for PbATG8 and qRT_atub1_f and qRT_atub1_r for α-tubulin 1 (see Table S1 in the supplemental material). A quantitative PCR (qPCR) was run in the Applied Biosystems StepOnePlus PCR system. Values of PbATG8 and *P. berghei* α-tubulin 1 were determined, and their relative expression was compared to the α-tubulin 1 housekeeping gene of *P. berghei*.

### Microscopy techniques.

Immunofluorescence assays (IFAs) on *P. berghei*-infected hepatic cells or *P. falciparum* infecting a liver from a SCID Alb-uPA hu Hep mouse (humanized mouse) were performed as previously described (10, 19). Images were viewed with either (i) a Nikon Plan Apo 100× objective using a Nikon Eclipse 90i microscope (Nikon Instruments, Inc.) with pictures taken using a Hamamatsu ORCA-ER camera (Hamamatsu Photonics) or (ii) a Zeiss Plan Apo 100× objective using a Zeiss Axio Imager M2 microscope, pictures were captured using a Hamamatsu ORCA-R2 camera, and all pictures were analyzed with Volocity (version 6.3). Images were processed using iterative restoration (confidence limit 98% and iteration limit 25), and further processing was performed using Adobe Photoshop software (Adobe Systems Inc.). To measure the association between proteins, PCC and the positive PDM images were calculated using Volocity software. An association was considered small for PCC values from 0.1 to 0.3, medium (i.e., restricted to some area) for values from 0.3 to 0.5 and strong for values from 0.5 to 1.0. To compare the amounts of PbATG8 or ACP expressed by the mutant, parental, and wild-type strains, the intensity of the fluorescent signal on images captured at the same exposure time and at the same gain, and offset values was analyzed by sampling several regions taken from 12 z-slices (spacing of 0.1 µm) within each PV using a region of interest of fixed size. The mean intensity of the fluorescence signal was calculated and subtracted from the mean background fluorescence intensity (three regions located between cells on the same slide) and expressed as corrected total cell fluorescence values [CTCF = integrated density − (area of selected cell × mean fluorescence of background readings)]. To determine the size of the PV and merosomes of the mutant and parental strains, the perimeter of PV or merosomes was delineated, at their largest circumference based on the fluorescence intensity of the Hsp70 signal, using the “region of interest” tool and the area was calculated using Volocity software. For thin-section transmission EM, *P. berghei*-infected Hepa 1-6 cells or pellets of *P. berghei* merosomes were fixed in 2.5% glutaraldehyde (Electron Microscopy Sciences, Hatfield, PA) in 0.1 M sodium cacodylate buffer (pH 7.4) for 1 h at room temperature and processed as described previously (3) before examination with a Philips CM120 electron microscope (Eindhoven, the Netherlands) under 80 kV.

## SUPPLEMENTAL MATERIAL

Table S1  List of primers used in this study Table S1, DOCX file, 0.1 MB
