# Supplementary material for: Overexpression of Plasmodium berghei ATG8 by Liver Forms Leads to Cumulative Defects in Organelle Dynamics and to Generation of Noninfectious Merozoites
Source: mBio. 2016 Jun 28;7(3):e00682-16. doi: 10.1128/mBio.00682-16 (PMC4937212; doi:10.1128/mBio.00682-16)
Supplement: Table S1 — List of primers used in this study [file mbo003162873st1.docx]

Table S1: List of primers used in this study

UTR-Sph1-r 5’-GCATATGCATGCGCATATATATACGCC-3’

UTR-HindIII-FRT-f 5’-CGAGGAAGCTTGAAGTTCCTATTCTCTAGAAAGTATAGGAACTTCAAA TAAAAAGTGGTAACTTTTTTTATACAG-3’

c-term-Not1-r 5’-CAGGCGAGCGGCCGCTGTTTATTAAAATTATCCAAAACAACTTTCAC-3’

c-term-Sph1-f 5’-GCATATGCATGCGATATTGTTTGGAAAATTTCG-3’

Ef1alpha-r 5’-ACCATGGATCCCCCTATG-3’

PbAtg8-304-f 5’-CTTAAAGGATAGTATGTGTGCG-3’

PbAtg8+750-r 5’-CATAAGCGATCATGAAAATATCC-3’

coko-f 5’-GATGTGCTGCAAGGCGATTAAG-3’

PbHsp70-r 5’-ACTTCAATTTGTGGAACA-3’

PbHsp70-f 5’-TGCAGCAGATAATCAAACTC-3’

PbGRASP-r 5’-CTGCCGAGCATTCATCATTAACATG-3’

PbGRASP-f 5’-GAAGGGGTTCGAATTTTAAAGCCG-3’

PbVPS4-811-r 5’-TCTTCGCCTGAAACCACTATC-3’

PbVPS4-453-f 5’-ATTATATGGCCCACCAGGAAC-3’

PbGADPH-r 5'-CACCAGTTGAAGCTGGGATAA-3'

PbGADPH-f 5'-GGACCATCAAAGGGAGGTAAAG-3'

PbαTub-r 5’-CGTGGATATGGTACTAAATTAGTTTGAAATTCGG-3’

PbαTub-f 5’-CTGGAAAGGAAGACGCAGCAAAC-3’

qRT_8_r 5’-TTCACCCACTAACATATTCATAGGG-3’

qRT_8_f 5’-TGCAGAAACTCATAAAATTCGAGC-3’

qRT_atub1_r 5’-TGGGCACCAATCAACAAATTG-3’

qRT_atub1_f 5’-GATGGCAAAATGTGATCCTAGAC-3’
